# Outcomes of experimental rat varicocele with and without microsurgery

**DOI:** 10.1186/s12894-015-0012-y

**Published:** 2015-03-17

**Authors:** Tie Zhou, Huan Cao, Guanghua Chen, Bo Yang, Yinghao Sun

**Affiliations:** Department of Urology, Changhai Hospital, The Second Military Medical University, 168 Changhai Road, 200433 Shanghai, PR China; Department of Urology, Haining People’s Hospital, 2 QianJiang West Road, 314400 Haining City, ZheJiang Province PR China

**Keywords:** Varicocele, Microsurgery, Rat

## Abstract

**Background:**

Experimental rat varicocele was usually developed by the conventional technique but with varied success; and microsurgical rat varicocele model was an effective alternative. In this study we further analyzed differential outcome of experimental rat model with and without microsurgery.

**Methods:**

One hundred and twenty male Sprague-Dawley rats were randomly assigned to two groups. In Group A, experimental rat varicocele model was developed with conventional technique. The left renal vein was partially ligated with concurrent ligation of communicating branches between the left spermatic vein and common iliac vein. In Group B, all the above procedures were finished with microsurgical manipulation under operating microscope. Before and after model development, the mean diameter of the left internal spermatic vein was compared; and at 8 weeks after initial surgery the mean sperm concentration and motility in both groups was analyzed.

**Results:**

The baseline mean diameter of the left internal spermatic vein in Group A and Group B was 0.14 ± 0.04 and 0.15 ± 0.03 mm, respectively (P =0.3157). In Group A 9 rats had severe complications resulting in model failure; while in Group B all rats had successful model except for one died of anesthetic accident (P = 0.008). At 8 weeks after initial surgery the mean left internal spermatic vein, sperm concentration and motility in both groups was 1.65 mm, 321.5×10^6^/gm and 51.9%; and 1.65 mm, 318.9×10^6^/gm and 53.5% respectively. There was nonsignificant difference of internal spermatic vein diameter, sperm concentration and motility between two groups.

**Conclusions:**

Microsurgery makes developing experiment rat varicocele model easy. Compared with conventional technique, microsurgical rat varicocele model has higher success rate and less complication.

## Background

Varicocele is believed to be associated with subfertility and has been found in 15% of normal male population [1]. Also, varicocele is an underlying cause in 41% of male patients with primary infertility and 75-81% of male patients with secondary infertility [2]. Although testicular hypoxia, hormonal dysfunction, elevated testicular temperature and spermatic veins hypertension have been considered to be involved in varicocele-related testicular dysfunction [1,3], the exact pathophysiologic mechanism is not yet completely understood.

Experimental rat varicocele is the most common animal model to investigate the molecular mechanism of varicocele induced male infertility. But developing rat model with the conventional technique has varied success [4-6]. Recently microsurgical rat varicocele model has been considered to be effective in dilation of spermatic vein and reduction of sperm concentration as well as motility [7]. In this study we further analyzed differential outcome of experimental rat model with and without microsurgery.

## Methods

### Study design

One hundred and twenty male Sprague-Dawley rats weighing 250-300 g were selected and randomly assigned to Group A (conventional technique) and Group B (microsurgery technique) with the method of random digits table. A sample size of 60 rats per group was sufficient according to the calculation formula when 80% power and a = 0.05 were considered. The study was approved by the Animal Care and Ethics Committee of SHANGHAI CHANGHAI hospital. Animals were housed under standard conditions in controlled environment with free access to food and water under a 12-hour day/night cycle.

### Surgery

1. Experimental rat varicocele model with conventional technique (Group A)

The model was induced according to the procedure described by Saypol and associates [8], with minor modifications. After 12 hours fasting, the rat was anesthetized by pentobarbital sodium (50 mg/kg) through intraperitoneal injection. The rat was fixed in supine position and the abdominal cavity was entered through a midline laparotomy incision. The abdominal contents were pushed to the right to identify the left kidney, the left renal vein and the left spermatic vein. The external diameter of the left internal spermatic vein was measured using micrometers at level of crossing iliolumbar vein. A metal probe 0.85 mm in diameter was placed on the left renal vein and a 4-0 silk suture tied around the vein and metal probe, medial to the adrenal and internal spermatic veins. The probe was removed, and the vein expanded against the limit of the suture loop. Subsequently, communicating branches between the left spermatic vein and common iliac vein were dissected and fully ligated using a 4-0 silk suture. Finally, the midline incision was closed in two layers with 3-0 silk suture. Eight weeks after initial surgery, the rat was anesthetized and a midline abdominal incision was again made. The external diameter of the left internal spermatic vein was measured again at the similar level to that of initial surgery. All measurements were confirmed by 2 investigators.

2. Experimental rat varicocele model with microsurgery (Group B)

**Figure 1:**
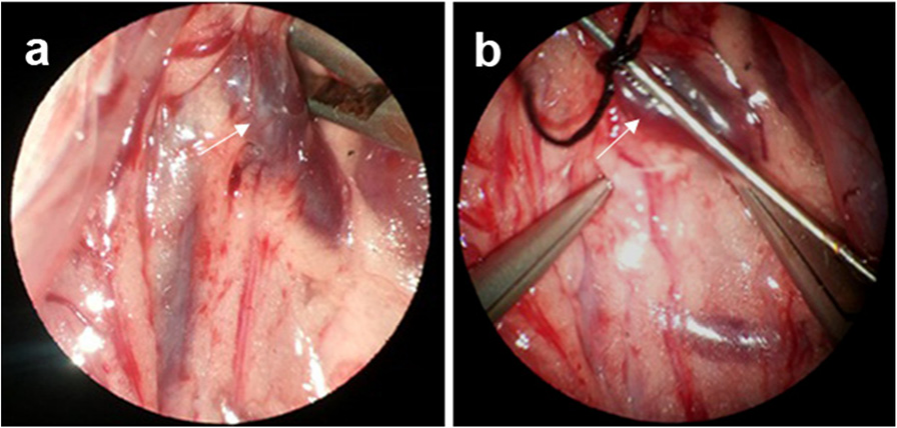
**Under enlarged field by operation microscopy, the tunnel around the renal vein was easily dissected (a), and partial ligation of left renal vein was easily performed (b).** Arrow: renal vein.

**Figure 2:**
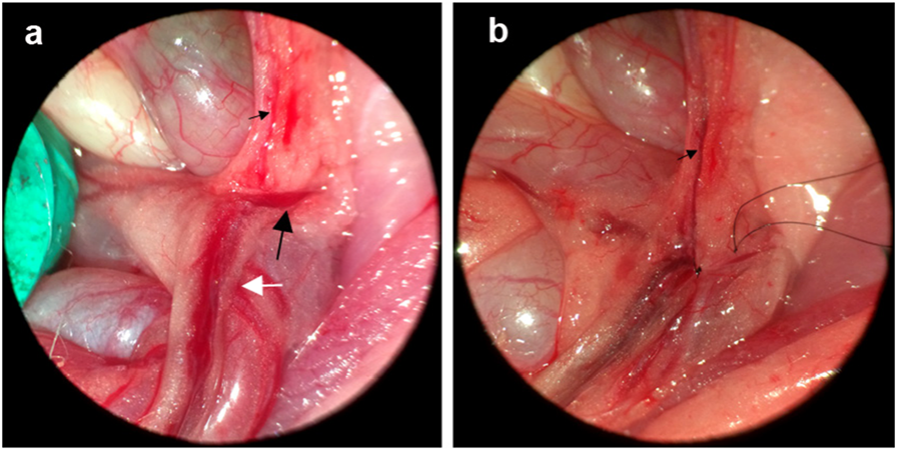
**Under operation microscope, the left internal spermatic vein with branch to the common iliac vein and the adjacent ureter were clearly identified (a); and the branch to the common iliac vein was ligated with 10-zero nylon (b).** Small black arrow: internal spermatic vein; big black arrow: branch to the common iliac vein; big white arrow: ureter.

Similarly, the rat was anesthetized by pentobarbital sodium (50 mg/kg) and the midline incision was made. The following manipulation was performed under operating microscope with 16× (Yi Guang Instrument company, Shanghai, China). Under magnification, the tunnel around the renal vein was easily dissected, and partial ligation of left renal vein was easily performed (Figure 1). Also, the left internal spermatic vein with branch to the common iliac vein and the adjacent ureter were clearly identified (Figure 2a). The branch to the common iliac vein was ligated with 10-zero nylon (Figure 2b). The remainder of the procedure was identical to that of Group A.

### Sperm evaluation

The cauda epididymis from each testis was weighed and minced in 5 ml of the media (Hank’s solution containing 0.5% bovine serum albumin) at 37°C. Solution was then placed on a slide glass that was warmed to 37°C for observation of sperm motility. Sperm motility was determined by counting >200 spermatozoa in randomly selected fields under a light microscope. The sperm count was calculated as the number of spermatozoa per gram of cauda epididymis. Sperm concentration and motility were evaluated by two licensed clinical andrology laboratory technologist blinded to the specimen group simultaneously and the average value was adopted.

### Statistical analysis

We calculated descriptive statistics for each variable. Before analysis we examined each variable for its distributional characteristics. All data are shown as the mean ± SD. Statistical significance was defined as P < 0.05 for a two-tailed test. Calculation was done using SAS® 9.1.

## Results

The mean baseline total body weight of rats in Group A and Group B was 274.8 ± 13.1 and 271.4 ± 12.4 gm, respectively (P = 0.1432). Baseline mean diameter of the internal spermatic vein in Group A and Group B was 0.14 ± 0.04 and 0.15 ± 0.03 mm, respectively (P =0.3157).

In Group A 9 rats had severe complications resulting in model failure; while in Group B no severe complication happened, and all rats had successful model except for one died of anesthetic accident (85% VS 98.3%, p = 0.008). Of 9 rats in Group A with model failure, 3 rats had injury of common iliac vein and 2 rats had injury of left renal vein immediately at developing the model. At eight weeks after surgery, another 4 rats’ left internal spermatic vein could not be identified because of adhesion from pyonephrosis (3 rats) or abdomen abscess (1 rat).

At 8 weeks after initial surgery the mean internal spermatic vein diameter, sperm concentration and motility in both groups were shown in Table 1. There was nonsignificant difference of internal spermatic vein diameter, sperm concentration and motility between two groups.

**Table 1:** **The mean diameter of internal spermatic vein and sperm concentration and motility in both groups at 8 weeks after surgery**

**Group (n)**	**Diameter of internal spermatic vein (mm)**	**Sperm**	
		**Concentration (10** ^**6**^ **/gm)**	**Motility (%)**
A (51)	1.65 ± 0.29	321.5 ± 19.9	51.9 ± 4.8
B (59)	1.65 ± 0.25	318.9 ± 13.6	53.5 ± 5.5
P value	0.9465	0.4076	0.1114

## Discussion

Varicocele has been considered to be closely associated with male infertility, but involved mechanism is not yet completely understood. Difficult tissue acquisition from human, forbidden invasive experiment in human and indefinite patient characteristics made the mechanism study in human is impossible. Thus animal models have been playing an important role in studying pathophysiology of varicocele.

Initially Kay and associates induced varicocele in rhesus monkey by partially ligating the left renal vein [9]. The first animal model reported decreased sperm counts and bilateral elevated testicular temperature. Harisson and co-workers conducted a similar animal model and reported bilateral impairment of lymphatic drainage and decreased testicular blood flow [10]. Subsequently Saypol et al extended the monkey model to dogs, followed by Cockett et al. and Dandia et al. [8,11,12] But in above animal models the reversion of abnormalities in semen characteristics and reduction of dilation degree in spermatic vein has occurred. Also, in 1981 Saypol et al introduced rat experimental model by partially ligating the left renal vein [8]. Since then experimental rat varicocele has become the most common animal model because of similar venous anatomy between human and rat when left varicocele occurs.

Even so, vascular variation adds indefinite factors to successful development of rat varicocele model. Similar to human anatomy, experimental rat varicocele accompanies dilation of the pampiniform plexus, the spermatic vein, and the collaterals leading to the iliac. In most rats more thinner internal spermatic vein from the pampiniform plexus drains into the left renal vein while more thicker branch vein into left common iliac. This pelvic venous drainage can negate the increased venous pressure proximal to partial occlusion of the left renal vein. Thus in this study we ligated the branch veins to the left common iliac besides partially ligating left renal vein. Such a modification has been demonstrated to be important for success of varicocele induction in rats by Turner et al and Najari et al. [7,13]

Although experimental rat varicocele model is widely used, rare report has referred to the complications of developing rat varicocele model. In this study we found success rate of rat varicocele model with conventional technique is only 85%. Except for variation of veinous anatomy, complication may be also the main factor. Vascular injury and pyonephrosis are the most common complications. Vascular injury often happens just when ligating the left renal vein or branches to left common iliac vein. Inadvertent puncture or tear of the vein from blind dissection behind the vein is the possible reason. Pyonephrosis is mainly ascribed to accident ligation of ureter adjacent to spermatic vein, followed by obstructive hydronephrosis and infection.

Comparison of the mean left internal spermatic vein diameter and sperm parameters between two groups shows no significant difference, but there was less complication and higher success in Group B. Addition of microsurgery to conventional technique results in less invasiveness in developing model. Operating microscope with 16× magnification will provide clear and enlarged visual field; and, combined with microsurgical instruments, make the dissection of blood vessel, even tiny branch, easy and safe. Moreover, microsurgical modeling of rat varicocele can easily identify the ureter adjacent to left internal spermatic vein and make ligation of tiny branch to common iliac vein with 10-zero nylon possible, which avoids injuring the ureter.

There are some limitations in this study. Microsurgical rat model needs special training and instruments, which are not commonly used in laboratories. Although the effect of our models on sperm parameters has been referred to in this study, the models as the platform of studying infertility are still further evaluated in the future study.

## Conclusion

With operating microscope, developing experimental rat varicocele model becomes easy and safe. Compared with conventional technique, microsurgical rat varicocele model has high success rate and less complication.
